# suPAR is associated with risk of future acute surgery and post-operative mortality in acutely admitted medical patients

**DOI:** 10.1186/s13049-018-0478-1

**Published:** 2018-02-01

**Authors:** Jeppe Meyer, Morten Alstrup, Line J. H. Rasmussen, Martin Schultz, Steen Ladelund, Thomas H. Haupt, Jens Tingleff, Kasper Iversen, Jesper Eugen-Olsen

**Affiliations:** 1 Clinical Research Centre, Copenhagen University Hospital Amager and Hvidovre, 2650 Hvidovre, Denmark; 2 Department of Cardiology, Copenhagen University Hospital Herlev, 2730 Herlev, Denmark; 3 Acute Medical Department, Copenhagen University Hospital Amager and Hvidovre, 2650 Hvidovre, Denmark

**Keywords:** Biomarker, Risk prediction, suPAR, Soluble urokinase plasminogen activator receptor, Risk assessment, Acute surgery, Post-operative mortality

## Abstract

**Background:**

Acutely admitted medical patients are often fragile and in risk of future surgery. The biomarker soluble urokinase plasminogen activator receptor (suPAR) is a predictor of readmission and mortality in the acute care setting. We aimed to investigate if suPAR also predicts acute surgery, which is associated with higher mortality than elective surgery, and if it predicts post-operative mortality.

**Methods:**

A retrospective registry-based cohort study of 17,312 patients admitted to an acute medical unit in Denmark, from 18 November 2013 until 30 September 2015. The first admission with available suPAR was defined as the index admission, and patients were followed via national registries until 1 January 2016. The risk of acute surgery during the entire follow-up period as well as the 90-day post-operative mortality risk was modeled by Cox regression analyses adjusted for sex, age, C-reactive protein, and Charlson Comorbidity Index (Charlson Score).

**Results:**

Acute surgery was carried out on 2404 patients (13.9%) after a median of 45 days (interquartile range 5–186) following the index admission. Patients receiving acute surgery had higher baseline suPAR compared with patients receiving elective- or no surgery (*p* < 0.0001). The hazard ratio (HR) for acute surgery was 1.50 (95% confidence interval (CI): 1.42–1.59) for every doubling of the suPAR level in the adjusted Cox regression analysis. Death within 90 days occurred in 439 (18.3%) patients receiving acute surgery, and the adjusted HR for post-operative mortality was 1.73 (95% CI: 1.52–1.95).

**Discussion:**

Elevated levels of suPAR in acutely admitted medical patients were independently associated with increased risk of future acute surgery as well as with 90-day post-operative mortality.

**Trial registration:**

This retrospective registry-based cohort study was approved by the Danish Health and Medicines authority (reference no. 3–3013-1061/1). All processing of personal data followed national guidelines, and the project was approved by the Danish Data Protection Agency (reference no. HVH-2014-018, 02767).

## Background

Patients admitted for an acute medical condition (acute medical patients) are at high risk of readmission and mortality after discharge [1]. In addition, these patients may undergo acute surgery, either during the primary admission or later during a readmission. Acute surgery is associated with higher morbidity and mortality compared with elective surgery [2–4]. The reasons for the increased mortality are multifactorial, but in some cases acute surgery can be an expression of critical illness in patients with more advanced stages of disease [5]. Acute hospitalization creates an opportunity to identify patients at high risk of acute surgery. Early recognition of the need of surgery could reduce the number of surgical procedures performed under acute circumstances, which may in turn reduce associated morbidity and mortality. Furthermore, identification of patients who are at high risk of post-operative mortality may aid in the decision on when to operate and in planning resources for postoperative care.

Soluble urokinase plasminogen activator receptor (suPAR) is an unspecific marker of immune activation and reflects the overall inflammatory state of an individual. Elevated plasma levels of suPAR are strongly associated with development, presence, and progression of a broad range of diseases [6–8], such as cardiovascular diseases and diabetes [9–11], certain types of cancer [9, 12–14], chronic and acute kidney disease [15, 16], and infectious diseases [17–19].

Among acutely admitted medical patients, elevated suPAR is an independent risk marker associated with intensive care unit admissions, readmissions, and mortality [1, 20].

In this study, we aimed to investigate if suPAR measured at admission can predict the risk of future acute surgery and post-operative mortality following acute surgery in a large cohort of acutely admitted medical patients.

## Methods

### Study design and setting

This retrospective, registry-based study was carried out at Copenhagen University Hospital Amager and Hvidovre, Hvidovre, Denmark, investigating patients who were admitted and had blood samples drawn at the Acute Medical Unit (AMU) from 18 November 2013 until 30 September 2015. Since 18 November 2013, suPAR was routinely analyzed in all AMU patients as part of the standard blood samples taken at admission. Data on a subgroup of this population was previously described by Rasmussen et al. [1]. The index admission was defined as the first admission during this period where suPAR was measured. The patients were followed in national registries for minimum 90 days from the index admission through 31 December 2015. The Danish personal civil registration number (CPR number) was used to link patient data from national registries with laboratory data from the electronic hospital database LABKA. The Danish National Patient Registry (NPR) contains information on all hospital admissions, including dates of admission and discharge, registered diagnoses, and surgical procedures performed. Hospital admission contacts in the NPR with less than 5 h apart were considered to belong to the same hospital admission, while contacts with more than 5 h apart were considered to belong to separate admissions. The survival status was extracted from the Civil Registration System.

### Selection of participants

Patients were included if they had standard blood samples analyzed during their admission to the AMU. Children, obstetrical-, gynecological-, gastroenterological-, and acute surgical patients were admitted to specialized acute departments and were therefore not included. Patients were excluded if no suPAR measurement was available or if follow-up via national registries was not possible.

### Outcomes

The primary outcome was surgery, defined as a patient having any surgical procedure registered in the NPR during an admission in the follow-up period: acute surgery was defined as an admission containing at least one acute surgical procedure; elective surgery was defined as an admission containing only planned, and no acute, surgical procedures. Thus, if a patient had both an elective and an acute surgical procedure registered within the same admission, it was defined as acute surgery. The distinction between acute and elective surgical procedures was made based on a variable in NPR that indicates whether the admission for a given surgical contact is acute or planned. Surgical procedures were stratified according to specialty, using the classification from the Danish national Healthcare Classification System (SKS). Smaller surgical procedures, such as lumbar- and ascites puncture and needle biopsies, and investigative procedures, such as endoscopies, were included in the analyses as surgery. A separate analysis excluding patients who only had minor surgery or endoscopies was carried out as well. Surgery types with less than 50 cases were compiled into the category “Other” (endocrine-, ophthalmological-, head and neck-, oral-, breast-, and tissue transplantation surgical procedures). Cancer-related surgery was defined as any surgical procedure for the primary diagnosis cancer (ICD-10 C00-C97). It should be noted that a patient can be in more than one surgical specialty.

The secondary outcome was all-cause mortality. When comparing mortality between non-surgical and surgical patients, we used mortality rates per person-year during the entire follow-up period. Patients who received surgery were categorized as non-surgical patients until the day of surgery; after that their time at risk was calculated for acute or elective surgery, respectively, until death or end of follow-up. This was done to prevent immortality bias. When comparing mortality between surgical specialties, we used 90-day post-operative mortality calculated from the first day of surgery.

### Measurements

Plasma levels of suPAR were analyzed as part of the standard blood samples at the Department of Clinical Biochemistry as previously described [1]. Briefly, suPAR was measured daily during weekdays in singlets using the suPARnostic**®** AUTO Flex ELISA on a Siemens BEP2000 analyzer according to the manufacturer’s instructions (ViroGates A/S, Birkerød, Denmark). C-reactive protein (CRP) was measured using a COBAS 6000 analyzer (Roche Diagnostics, Mannheim, Germany).

### Charlson score

The Charlson Comorbidity Index (Charlson score) [21], was calculated using all diagnoses registered in the NPR for the index admission and in the 2 years prior to the index admission. The Charlson Score is a weighted scoring system, rating the number and severity of chronic comorbidities, including chronic kidney disease among others. We used a SAS macro (developed by Ken Turner and Charles Burchill) based on ICD-10 diagnoses to calculate the Charlson score [22], using the revised weighting by Quan et al. [23].

### Data analysis

Continuous data are presented as medians with interquartile range (IQR). Differences in continuous variables were tested by Student’s *t*-test or Wilcoxon’s two-sample test, and categorical variables were tested by Chi-squared test. The correlation between suPAR and CRP was calculated using Kendall rank correlation coefficient.

The risk of acute surgery and post-operative mortality was modeled by Cox regression as a function of log2-transformed suPAR adjusted for sex, age, CRP, and Charlson score. A second analysis was made including creatinine in the adjustment. We used a combined endpoint for acute surgery, using death and elective surgery as competing risks. In the Cox regression analysis of post-operative mortality, the number of days to surgery was also included as an explanatory variable to adjust for differences in time from suPAR measurement to surgery. We also tested for an interaction between the suPAR level and time from suPAR analysis to surgery. The results are presented as hazard ratios (HRs) with 95% confidence intervals (CIs).

The risk of acute surgery and 90-day post-operative mortality according to suPAR level was presented in cumulative incidence plots and Kaplan-Meier plots, respectively. suPAR values were stratified in 3 ng/ml intervals to ensure that the results were less dependent on the specific cohort and thereby comparable with other patient cohorts. The log-rank test was used to compare mortality or risk of acute surgery between suPAR strata in the cumulative incidence and Kaplan-Meier plots.

SAS Enterprise Guide 7.1 (SAS Institute, Cary, North Carolina, USA) and R 3.0.3 (R Foundation for Statistical Computing, Vienna, Austria) were used for statistical analysis. A *p*-value < 0.05 was considered statistically significant.

## Results

### Participants

**Fig. 1:**
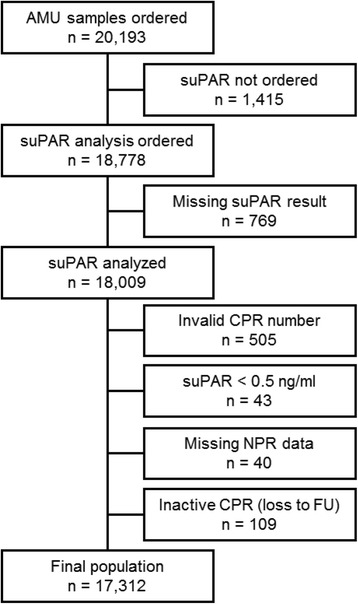
Flow diagram of included patients. AMU, Acute Medical Unit; CPR, civil registration number; NPR, National Patient Registry, suPAR, soluble urokinase plasminogen activator receptor

The cohort consisted of 20,193 patients who were admitted to the AMU for an acute medical condition. A total of 2881 were excluded (Fig. 1), leaving 17,312 patients for analysis. Baseline characteristics are shown in Table 1. The median follow-up time for all included patients was 394 days (IQR 218–583). During follow-up, 3768 (21.8%) patients had surgical procedures performed. Of these, 2404 (63.8%) were acute (Table 2). The median time from suPAR measurement at the index admission until surgery was 74 days (IQR 9–218); 45 days (IQR 5–186) for acute surgery and 118 days (IQR 37–275) for elective surgery.

**Table 1:** Baseline characteristics of acutely admitted medical patients at the index admission

Characteristics	Patients (*n* = 17,312)
Male sex, n (%)	8118 (47)
Age (years), median (IQR)	61.3 (43.3–76.3)
CRP (mg/l), median (IQR)	5 (1–31)
suPAR (ng/ml), median (IQR)	2.8 (1.9–4.3)
Charlson score, median (IQR)	0 (0–1)

*CRP* C-reactive protein, *IQR* interquartile range, *suPAR* soluble urokinase plasminogen activator receptor

**Table 2:** Acutely admitted medical patients stratified in relation to surgery

Characteristics	No surgery (*n* = 13,544)	Acute surgery (*n* = 2404)	*p-*value^a^	Elective surgery (*n* = 1364)	*p-*value^b^
Male sex, n (%)	6308 (46.6)	1113 (46.3)	0.80	697 (51.1)	0.0014
Age, median (IQR)	60.0 (41.8–76.1)	66.4 (47.4–78.8)	< 0.0001	64.9 (51.7–74.5)	< 0.0001
CRP, median (IQR)	5 (1–26)	12 (3–61)	< 0.0001	5 (1–22)	0.30
suPAR, median (IQR)	2.7 (1.9–4.0)	3.6 (2.4–5.7)	< 0.0001	2.9 (2.1–4.2)	< 0.0001
Charlson score, median (IQR)	0 (0–1)	0 (0–2)	< 0.0001	0 (0–1)	< 0.0001
Mortality rate per person-year	0.12	0.37	< 0.0001	0.13	0.60

*CRP* C-reactive protein, *IQR* interquartile range, *suPAR* soluble urokinase plasminogen activator receptor

^a^Acute surgery compared to no surgery

^b^Elective surgery compared to no surgery

### suPAR is associated with acute surgery

Patients receiving acute surgery were older and had higher suPAR and CRP levels at the index admission compared with non-surgical patients (Table 2). Furthermore, the median Charlson score was higher, showing an increased prevalence of comorbidities among acute surgical patients (Table 2). Similarly, patients who underwent elective surgery were older and had higher suPAR and Charlson score compared with non-surgical patients (Table 2). For the entire population, suPAR and CRP were positively correlated (Kendall’s tau b 0.36, *p* < 0.0001).

In the Cox regression model adjusted for sex, age, CRP, and Charlson score, suPAR was associated with acute surgery during follow-up, with a HR of 1.50 (95% CI: 1.42–1.59, *p* < 0.0001) for every doubling in suPAR. When including creatinine in the adjustment, the HR was 1.45 (95% CI: 1.37–1.54, *p* < 0.0001).

**Fig. 2:**
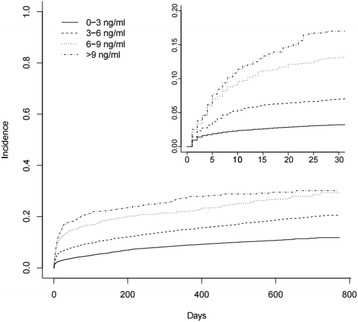
Cumulative incidence plot of patients’ risk of acute surgery stratified by suPAR concentrations. Competing risks are death and elective surgery. Inset: zoom on the first 30-day interval is shown. suPAR 0–3 ng/ml included 9720 (56.2%) patients, suPAR 3–6 ng/ml included 5537 (32.0%) patients, suPAR 6–9 ng/ml included 1299 (7.5%) patients, and suPAR >9 ng/ml included 756 (4.4%) patients. Log-rank test showed *p* < 0.0001. suPAR, soluble urokinase plasminogen activator receptor

Furthermore, we carried out a sub-analysis excluding 761 patients that exclusively had minor surgical procedures performed (minor surgery and endoscopies). In this sub-analysis, adjusted for sex, age, CRP and Charlson score, every doubling in suPAR was associated with a HR of 1.37 (95% CI: 1.29–1.47, *p* < 0.0001). Finally, the cumulative incidence rate of acute surgery increased with increasing suPAR (Fig. 2).

### suPAR in acute and elective surgery

When comparing acute with elective surgery, suPAR was increased in patients undergoing endoscopies, gastroenterological-, orthopedic-, respiratory-, urological- and, to a lesser degree, dermatological- and cancer-related surgical procedures (Table 3).

**Table 3:** Acute and elective surgery in individual specialties

	Acute surgery			Elective surgery				
Type of surgery	n (%)^a^	Median suPAR	90-days mortality: n (%)	n	Median suPAR	90-days mortality: n (%)	*p-*value suPAR^b^	*p-*value mortality^c^
Gastroenterological	353 (68.7)	3.7	75 (21.3)	161	3.2	9 (5.6)	0.0032	< 0.0001
Orthopedic	320 (57.7)	4.3	61 (19.1)	235	2.8	5 (2.1)	< 0.0001	< 0.0001
Cardiothoracic	111 (30.3)	2.8	8 (7.2)	255	2.6	3 (1.2)	0.16	0.0041^d^
Neurological	68 (44.7)	2.9	8 (11.8)	84	2.8	2 (2.4)	0.90	0.043^d^
Respiratory	357 (86.9)	4.4	131 (36.7)	54	3.1	5 (9.3)	< 0.0001	< 0.0001
Urological	87 (41.6)	4.4	14 (16.1)	122	3.1	5 (4.1)	< 0.0001	0.0029
Minor	381 (87.0)	4.5	114 (29.9)	57	3.8	4 (7.0)	0.065	0.0003
Endoscopies	764 (76.7)	4.2	145 (19.0)	230	3.3	11 (4.8)	< 0.0001	< 0.0001
Gynecological	34 (34.7)	2.8	1 (2.9)	64	2.7	0	0.53	0.17
Obstetrics	231 (89.1)	1.6	0	28	1.8	0	0.50	
Vascular	54 (36.7)	4.1	12 (22.2)	93	4.2	1 (1.1)	0.96	< 0.0001
Dermatological	161 (80.5)	4.1	29 (18.0)	39	3.7	1 (2.6)	0.022	0.0153^d^
Other	10 (10.5)	2.5	1 (10.0)	85	2.8	2 (2.4)	0.71	0.19
Cancer-related^e^	104 (40.5)	4.1	46 (44.2)	153	3.4	11 (7.2)	0.010	< 0.0001

*suPAR* soluble urokinase plasminogen activator receptor

^a^The percentage of total surgery in the specific specialty

^b^*p*-value is calculated for suPAR in acutely vs. electively operated patients

^c^*p*-value is calculated for acute 90-day mortality compared with elective 90-day mortality

^d^Fisher’s exact test is used instead of Pearson’s Chi-squared test

^e^Cancer-related surgery is measured as the patient having cancer as the main diagnosis when surgery was performed

### Mortality rates in acute medical patients

In the entire population of acutely admitted medical patients, mortality rates were higher among those who received acute surgery during follow-up compared with non-surgical patients, whereas mortality rates were similar among patients receiving elective surgery compared with the non-surgical patients (Table 2).

90-day post-operative mortality was higher in patients receiving acute surgery compared to elective in the majority of specialties (Table 3). This increased mortality was particularly evident for gastroenterological-, orthopedic-, respiratory-, vascular-, and cancer-related surgery as well as endoscopies, but also observed for cardiothoracic-, neurological-, urological-, minor-, and dermatological surgical procedures (Table 3).

### suPAR is associated with post-operative mortality in acutely operated patients

**Fig. 3:**
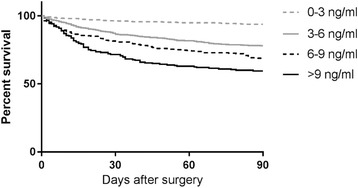
Kaplan-Meier plot of 90-day post-operative mortality in acutely operated patients, stratified on suPAR concentrations. suPAR 0–3 ng/ml included 955 (39.7%) patients, suPAR 3–6 included 921 (38.3%) patients, suPAR 6–9 included 320 (13.3%) patients, and suPAR >9 included 208 (8.7%) patients. Log-rank test showed *p* < 0.0001. suPAR, soluble urokinase plasminogen activator receptor

Of the patients receiving acute surgery, 439 died (18.3%) within 90 days follow-up after surgery. In Cox regression analysis adjusted for sex, age, CRP, and Charlson score, the HR for 90-day post-operative mortality for doubling the suPAR value was 1.73 (95% CI: 1.52–1.95, *p* < 0.0001). Due to variability in time from the index admission to the acute surgical procedure, we tested for an interaction between the suPAR level and time from suPAR analysis to surgery in the Cox regression analysis for 90-day post-operative mortality, and found no interaction (*p* = 0.86). The 90-day post-operative mortality after acute surgery was higher for patients with elevated baseline levels of suPAR (Fig. 3).

## Discussion

In this large cohort of acute medical patients, 14% underwent acute surgery during a median follow-up of little more than a year. Elevated suPAR levels were significantly associated with increased risk of future acute surgery as well as postoperative mortality when adjusted for sex, age, CRP, and comorbidity burden. To our knowledge, this study is the first to investigate an association between suPAR in patients admitted with an acute medical condition and the risk of receiving future acute surgery.

suPAR is a prognostic biomarker routinely measured in acute medial patients in our hospital. It has previously been shown that elevated suPAR level is associated with increased risk of admission to the intensive care unit, readmissions, and mortality in these patients [1, 8, 20]. In this study, we add the risk of future acute surgery to the list of negative outcomes associated with elevated suPAR level.

There is a limited amount of research that has used acute surgery as an outcome measurement, and the use of biomarkers for risk prediction in surgery is generally directed towards predicting post-operative outcomes, such as morbidity and mortality [24, 25]. A British study found associations between increasing CRP levels during admittance and the risk of undergoing acute surgery in patients presenting with acute renal colic. However, there was no association between the initial admission-level of CRP and the risk of acute surgery [26].

In contrast, we found that the initial suPAR level was associated with the risk of future acute surgery in an unspecific cohort of acute medical patients. This knowledge of the relation between suPAR and future acute surgery cannot be used to exactly predict which acute medical patients will undergo acute surgery, but it adds acute surgery as an adverse event to be aware of, when encountering patients with elevated levels of suPAR.

Elevated suPAR at the index admission was also found to be associated with future post-operative mortality. The mortality rate was higher in patients receiving acute surgery compared with both patients receiving elective and no surgery. To support this, earlier studies also showed increased mortality among acutely operated patients compared with elective patients [2–4]. Similarly, preoperative suPAR levels was shown to be an independent predictor of mortality in patients operated for colorectal cancer [27]. The observation that suPAR carries information on post-operative mortality suggests that suPAR could potentially supplement preoperative risk scoring systems, such as the American Society of Anaesthesiologists (ASA) score [28] or raise attention to high-risk patients prior to surgery.

In the cohort, the suPAR levels were significantly elevated among both patients receiving acute and elective surgery compared to patients receiving no surgery; the highest suPAR levels were observed in acutely operated patients. The acute surgical patients were more ill, as indicated by the presence of comorbidities and higher suPAR levels. Similarly, a large American study of surgical patients also observed a higher proportion of patients with preoperative systemic inflammatory response syndrome or sepsis and thereby higher preoperative inflammation level among acutely operated patients [3]. Even though there are differences between our study and the American study, both studies find that preoperative inflammation and comorbidities have an effect on the post-operative outcome [3].

suPAR was analyzed at a median of 74 days before surgery, indicating that it reflects an underlying pathology or frailty that increase the risk of acute surgery. It raises the question of whether suPAR measured immediately before surgery could have even stronger post-operative prognostic value. This was not possible to determine with the present study design.

Our study finds that suPAR can serve as an ‘attention marker’ for identifying the most fragile acute medical patients who have the highest risk of negative outcomes, including acute surgery.

## Limitations

This study has some limitations. The study was carried out at a single hospital and should be reproduced in a multicenter study to improve general applicability. In our hospital, acutely admitted gastroenterological patients, children, and obstetric patients are admitted through specialized acute departments, where suPAR is not routinely analyzed; therefore, these patients were not included in the study. Another limitation is the lack of data on smoking habits, which has been shown to increase suPAR levels with up to 1–2 ng/ml in the general population [29].

The types of surgery could have been stratified into risk levels in relation to post-operative mortality as the specific type of surgery has a large influence on post-operative mortality [30] and may be a potential confounder. A second analysis excluding minor surgery (ascites puncture, needle biopsies etc.) and endoscopies showed that the prognostic effect of suPAR is not limited to minor surgical procedures.

## Conclusion

In summary, we found that elevated suPAR levels in acute medical patients were associated with increased risk of future acute surgery and of post-operative mortality. The suPAR biomarker may, along with other biomarkers and clinical observations, aid the physicians in identifying acute medical patients in high risk of negative outcomes.

## Abbreviations

AMU: Acute medical unit
ASA: American Society of Anaesthesiologists
CI: Confidence interval
CPR: Civil registration number
CRP: C-reactive protein
HR: Hazard ratio
ICD: International Classification of Diseases
IQR: Interquartile range
NPR: National Patient Registry
SKS: Danish national Healthcare Classification System
suPAR: Soluble urokinase plasminogen activator receptor
